# Differential expression of ATP7A, ATP7B and CTR1 in adult rat dorsal root ganglion tissue

**DOI:** 10.1186/1744-8069-6-53

**Published:** 2010-09-13

**Authors:** Virginia Ip, Johnson J Liu, Julian FB Mercer, Mark J McKeage

**Affiliations:** 1 Department of Pharmacology and Clinical Pharmacology, School of Medical Sciences, Faculty of Medical and Health Sciences, The University of Auckland, Auckland, New Zealand; 2 Centre for Cellular and Molecular Biology, School of Life and Environmental Sciences, Deakin University, Melbourne, Australia

## Abstract

**Background:**

ATP7A, ATP7B and CTR1 are metal transporting proteins that control the cellular disposition of copper and platinum drugs, but their expression in dorsal root ganglion (DRG) tissue and their role in platinum-induced neurotoxicity are unknown. To investigate the DRG expression of ATP7A, ATP7B and CTR1, lumbar DRG and reference tissues were collected for real time quantitative PCR, RT-PCR, immunohistochemistry and Western blot analysis from healthy control adult rats or from animals treated with intraperitoneal oxaliplatin (1.85 mg/kg) or drug vehicle twice weekly for 8 weeks.

**Results:**

In DRG tissue from healthy control animals, ATP7A mRNA was clearly detectable at levels similar to those found in the brain and spinal cord, and intense ATP7A immunoreactivity was localised to the cytoplasm of cell bodies of smaller DRG neurons without staining of satellite cells, nerve fibres or co-localisation with phosphorylated heavy neurofilament subunit (pNF-H). High levels of CTR1 mRNA were detected in all tissues from healthy control animals, and strong CTR1 immunoreactivity was associated with plasma membranes and vesicular cytoplasmic structures of the cell bodies of larger-sized DRG neurons without co-localization with ATP7A. DRG neurons with strong expression of ATP7A or CTR1 had distinct cell body size profiles with minimal overlap between them. Oxaliplatin treatment did not alter the size profile of strongly ATP7A-immunoreactive neurons but significantly reduced the size profile of strongly CTR1-immunoreactive neurons. ATP7B mRNA was barely detectable, and no specific immunoreactivity for ATP7B was found, in DRG tissue from healthy control animals.

**Conclusions:**

In conclusion, adult rat DRG tissue exhibits a specific pattern of expression of copper transporters with distinct subsets of peripheral sensory neurons intensely expressing either ATP7A or CTR1, but not both or ATP7B. The neuron subtype-specific and largely non-overlapping distribution of ATP7A and CTR1 within rat DRG tissue may be required to support the potentially differing cuproenzyme requirements of distinct subsets of sensory neurons, and could influence the transport and neurotoxicity of oxaliplatin.

## Background

ATP7A, ATP7B and CTR1 are copper transporting proteins that have evolved along with other components of copper regulatory pathways for delivering copper to essential cuproenzymes without releasing highly cytotoxic free copper ions [1,2]. The P-type ATPases, ATP7A and ATP7B, both transport copper out of cells or into the trans-Golgi network [3], whereas CTR1 is a plasma membrane protein that functions as a high-affinity cellular copper uptake transporter [4]. ATP7A, ATP7B and CTR1 exhibit cell-type specific expression in the brain and other tissues [5,6], reflecting their requirements for copper to support the functions of diverse cuproenzymes, such as dopamine-β-monooxygenase and peptidylglycine α-amidating monooxygenase that convert dopamine to norepinephrine and synthesize neuropeptides, respectively [1]. Disturbance of copper transporters causes neurodegeneration. For example, mutation of ATP7A and ATP7B causes Menkes and Wilson disease, respectively, both of which have serious neurological sequelae including mental retardation, seizures, developmental delay and ataxia [7].

Little is currently known about the expression of copper transporters in the dorsal root ganglia (DRG) that contain the cell bodies of primary sensory neurons. These neurons may require copper transport as they strongly express cuproenzymes, such as cytochrome C oxidase [8], Cu/Zn superoxide dismutase [9] and peptidylglycine α-amidating monooxygenase [10], and are sensitive to copper deficiency [11,12]. In other cell types, copper transporters have been shown to have a role in controlling the cellular accumulation and cytotoxicity of platinum drugs, with CTR1 mediating platinum uptake into cells [13-15], and ATP7A and ATP7B transporting platinum out of cells or into specific sub-cellular compartments [16-20]. Platinum-based drugs, such as cisplatin and oxaliplatin, accumulate in DRG tissue [21-26], damage sensory neurons [21,22,24-33], and induce peripheral sensory neuropathies that limit their use in clinical cancer chemotherapy [34]. In the current study, we investigated the expression of ATP7A, ATP7B and CTR1 in DRG tissue from adult rats, either healthy control animals or those treated with oxaliplatin or its drug vehicle. Neuronal atrophy was used as the endpoint for measuring the neurotoxicity of oxaliplatin in DRG tissues, as in previous studies [26,29,35-37]. We aimed to determine patterns of expression and localization of ATP7A and ATP7B within DRG tissue, in an extension to our recent study of CTR1 [35], and to relate the expression of these copper transporters to the neurotoxicity of oxaliplatin.

## Results

### Copper transporter gene expression in DRG and other tissues

**Figure 1:**
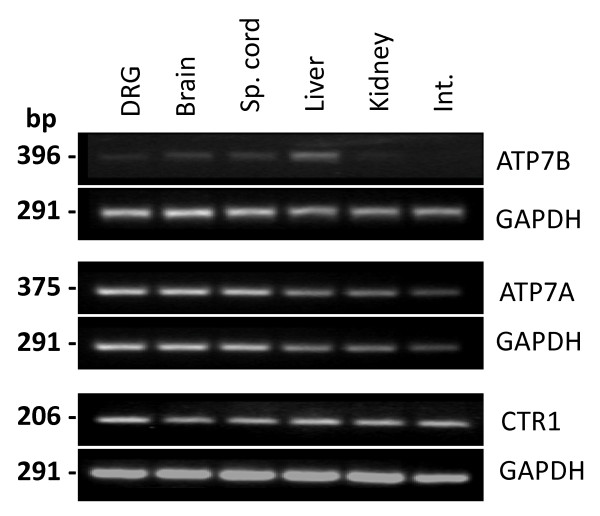
**mRNA expression of copper transporters in rat lumbar DRG and other tissues**. Representative gel electrophoresis bands of RT-PCR products for ATP7A, ATP7B, CTR1 and glyceroaldehyde-3-phosphate (GAPDH) genes in indicated tissues. Sp. Cord, spinal cord; Int., intestine.

The expression of copper transporter genes in rat DRG tissue was determined by RT-PCR and qPCR in comparison to reference tissues (brain, spinal cord, liver, kidney and intestine). The RT-PCR (Figure 1) and qPCR (Table 1) findings corresponded well with each other. In all of the tissues, CTR1 had the highest mRNA levels, followed by ATP7A and then ATP7B had the least, except in liver where ATP7B levels were higher than ATP7A.

**Table 1:** Copper transporter gene expression in rat tissues determined by quantitative PCR

	Copper transporter mRNA levels (2^(-DCT) ^× 10^4^) in indicated tissue					
	
	Dorsal Root Ganglion	Brain	Spinal Cord	Liver	Kidney	Intestine
ATP7B	0^d ^(0-1)	1^a ^(0-1)	1^b ^(0-1)	2 (1-6)	0^d ^(0-1)	0^d ^(0-1)
						
ATP7A	11 (7-44)	11 (7-84)	19 (5-289)	1^c ^(0-9)	2 (2-84)	5^a ^(0-43
						
CTR1	12 (4-323)	19 (4-38)	30 (2-81)	73 (11-275)	13 (6-243)	43 (7-353)

Values represent the median and range (in parenthesis) of determinations in six animals. Symbols indicate when gene expression was undetectable in one^a^, two^b^, three^c ^or four^d ^of six animals.

In DRG tissue, ATP7B mRNA was barely detectable with only faint or no bands visible on RT-PCR gels but in liver tissue bands were more clearly visible (Figure 1). ATP7B mRNA transcripts in DRG tissue were detected by qPCR in only two of six animals compared to all six animals for liver (Table 1). In DRG tissue, ATP7A mRNA was more readily detectable than ATP7B, with clearly visible bands on RT-PCR gels (Figure 1) and qPCR-detectable transcripts in all six animals (Table 1). ATP7A mRNA levels in DRG were similar to brain and spinal cord levels but higher than those in the non-neuronal reference tissues.

High levels of CTR1 mRNA were found in DRG, as in other tissues. RT-PCR gels showed clearly visible bands for CTR1 in all tissues (Figure 1). CTR1 mRNA transcripts was detectable by qPCR in all tissues, and in all animals, at levels higher than ATP7A and ATP7B (Table 1).

### Copper transporter protein expression in DRG tissue

**Figure 2:**
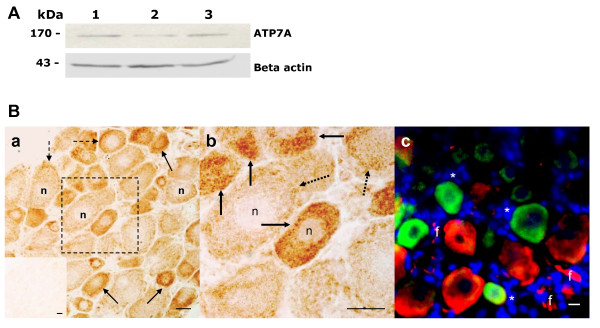
**ATP7A protein expression in rat DRG tissue**. (A) Detection of ATP7A protein by Western blot analysis in DRG of rats aged 4 weeks (lane 1), 12 weeks (lane 2) and 20 weeks (lane 3). Beta actin was probed as a loading control. (B) a and b, neuronal ATP7A immunoreactivity in rat L5 DRG tissue associated with cell bodies of small neurons, intense punctuate vesicular structures in cytoplasm (solid arrows), diffuse cytoplasmic and granular membrane staining (broken arrows), without staining of nuclei (n) or other tissue elements. b was the enlarged frame in a. Inset in a was a negative control. c, Fluorescent immunohistochemistry shows little overlap between ATP7A-immunoreactive (IR) neurons (green), phosphorylated neurofilament heavy subunit (pNF-H)-IR neurons (red), DAPI-stained satellite cells (asterisk) or nerve fibres (f). Scale bar, 20 μm.

ATP7A had a specific pattern of distribution within rat DRG tissue, with intense cytoplasmic staining of the cell bodies of smaller DRG neurons as revealed by immunohistochemistry. The specificity of anti-ATP7A primary antibody was confirmed by Western blotting showing a protein band with the size 170 kDa on ATP7A immunoblots of DRG tissue homogenates from rats aged 4, 12 and 20 weeks (Figure 2A). Negative controls that excluded the primary antibody lacked specific immunoreactivity (Figure 2B2a, inset). ATP7A immunohistochemistry of DRG tissue visualized by ABC-peroxidase revealed that this copper efflux transporter was most strongly expressed within smaller-sized DRG neurons that showed intense immunoreactivity in a punctuate pattern localised to the cytoplasm of their neuronal cell bodies (Figure 2B,a, and 2b). Other DRG neurons showed lighter and more diffuse cytoplasmic immunostaining for ATP7A, with occasional granular staining of the plasma membrane. No ATP7A immunoreactivity was apparent in the satellite cells, nerve fibres, or other non-neuronal tissue elements of the rat DRG. Furthermore, fluorescent immunohistochemistry showed that ATP7A immunoreactivity was mainly associated with smaller DRG neurons that did not overlap with DAPI-stained non-neuronal cells or with the pNF-H-immunoreactive larger neurons and nerve fibres (Figure 2B,c).

No specific immunoreactivity for ATP7B was found in rat DRG tissue when compared with a negative control (data not shown), even though the primary antibody (NB100-360, Novus Biologicals) detects ATP7B in rat liver [38].

**Figure 3:**
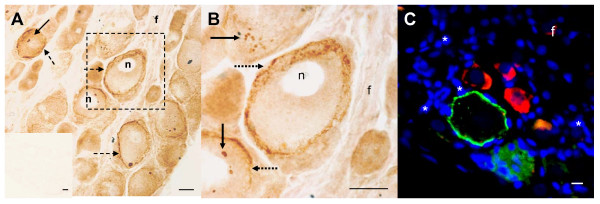
**CTR1 expression in rat DRG tissue**. (A) Neuronal CTR1 immunoreactivity in DRG tissue associated with the plasma membranes (broken arrow) and granular vesicular structures (solid arrow) of the cell bodies of large neurons, with lighter diffuse cytoplasmic staining of other neuronal cell bodies, without staining of nuclei (n) or nerve fibres (f). (B) Enlarged frame in A. Inset in A was a negative control. (C) Fluorescent immunohistochemistry shows no overlap between smaller-sized ATP7A-immunoreactive neurons (red) and larger-sized CTR1-immunoreactive neurons (green) or DAPI-stained satellite cells (asterisk). Scale bar, 20 μm.

CTR1 immunohistochemistry of rat DRG tissue showed a pattern of immunostaining that differed from ATP7A. The specificity of the Novus antibody for CTR1 immunohistchemistry has been previously determined by preabsorption assay with immunizing peptide using a hCTR1 A2780 human ovarian carcinoma cell line [39]. This antibody, however, is unsuitable for use in Western blot analysis. Strong CTR1 immunoreactivity was associated with the plasma membrane and cytoplasmic vesicular structures of larger-sized DRG neurons, whereas only light staining appeared in the remaining neurons (Figure 3A,B), as previously described [35]. Double label fluorescent immunohistochemistry provided further evidence of CTR1 and ATP7A primary localisation to neuronal cell bodies, and their distinct patterns of immunoreactivity and non-overlapping distribution within rat DRG tissue (Figure 3C).

### Morphometry of ATP7A-positive and CTR1-positive neurons in DRG tissue from control and oxaliplatin-treated rats

**Figure 4:**
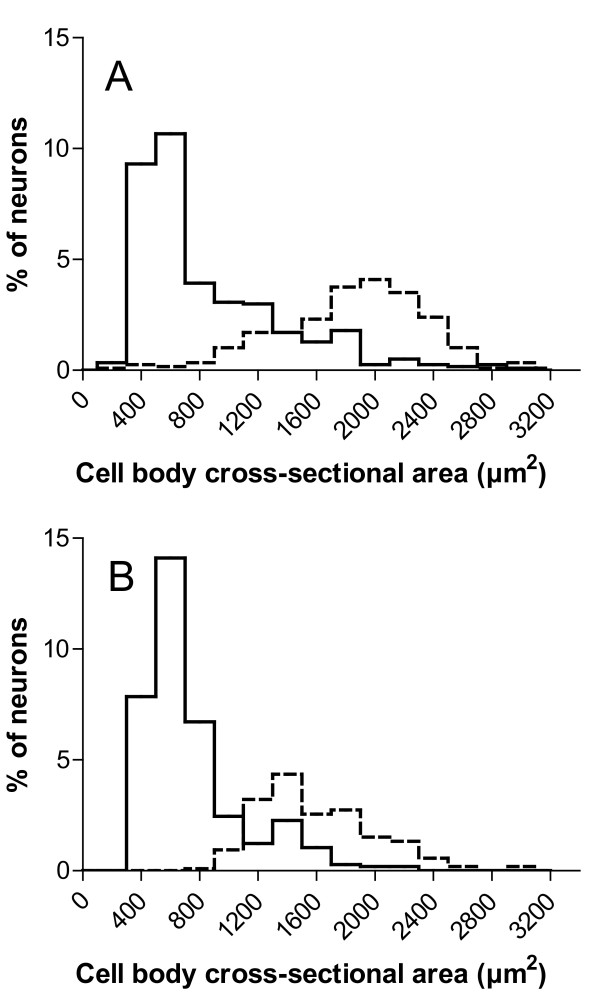
**Cell body size frequency histograms of neuronal cell bodies with strong expression for ATP7A (solid lines) and CTR1 (dotted lines) in DRG tissue**. ATP7A and CTR1 were strongly expressed distinctly by the small neurons and large DRG neurons, respectively, from control (A) and oxaliplatin-treated animals (B). Oxaliplatin caused a left-ward shift in the size profile of strongly CTR1-immunoreactive neurons but not that of strongly ATP7A-immunoreactive neurons. Each bin represents the mean value of 5 animals.

Morphometric analysis of control animal DRG tissue showed that ATP7A and CTR1 were expressed by different neuronal subpopulations with differing size profiles (Figure 4; Table 2). For this analysis, strongly ATP7A-expressing DRG neurons were defined as those having intense diffuse or punctuate cytoplasmic staining and/or plasma membrane immunoreactivity to ATP7A. Those negative for strong ATP7A expression had no or low-intensity diffuse or punctuate cytoplasmic staining without plasma membrane immunoreactivity. Strongly CTR1-expressing DRG neurons were defined as those having intense plasma membrane and/or punctuate cytoplasmic immunoreactivity to CTR1. Those negative for strong CTR1 expression had only diffuse cell body immunoreactivity without plasma membrane or punctuate cytoplasmic immunoreactivity. DRG neurons with strong expression of ATP7A accounted for 35.1 ± 2.9% of the overall population of DRG neurons in control animals, whereas those with strong expression of CTR1 accounted for 10.9 ± 1.8% (*P *< 0.001). About 64.2 ± 6.9% of the strongly ATP7A-expressing neurons had cell bodies measuring < 750 μm^2^, but only 2.0 ± 1.3% of strongly CTR1-expressing neurons were of this size (*P *< 0.001). About 58.2 ± 16.1% of strongly CTR1-expressing neurons had cell bodies measuring >1750 μm^2^, but only 6.7 ± 2.5% of strongly ATP7A-expressing neurons were of this size (*P *< 0.001). The mean cell body area of strongly ATP7A-expressing neurons (767.1 ± 87.6 μm^2^) was significantly smaller than that of the strongly CTR1-expressing neurons (1936 ± 278 μm^2^; *P *< 0.001).

**Table 2:** Morphometry of subpopulations of DRG neurons with strong immunoreactivity (IR) for ATP7A and CTR1 in tissues from control and oxaliplatin-treated animals

Animal Group	Copper transporter	Frequency of IR neurons (%)	Small-sized cells ( < 750 μm^2^) (%)	Medium-sized cells (750-1750 μm^2^) (%)	Large-sized cells ( > 1750 μm^2^) (%)	Mean cell body area (μm^2^)
Control	ATP7A	35.1 ± 2.9	64.2 ± 6.9	29.1 ± 4.7	6.7 ± 2.5	767 ± 88
	CTR1	10.9 ± 1.8^†^	2.0 ± 1.3^†^	39.9 ± 15.9	58.2 ± 16.1^†^	1936 ± 278^†^
	
Oxaliplatin treatment	ATP7A	33.6 ± 2.3	70.5 ± 4.0	27.1 ± 3.7	2.4 ± 1.3	641 ± 39
	CTR1'	11.3 ± 3.5	5.2 ± 2.0	66.4 ± 5.5*	28.5 ± 5.5*	1461 ± 64*

Values represent the mean ± standard deviation of determinations in 4 to 6 animals per group. ^† ^*P *< 0.01 for comparison with ATP7a control group; * *P *< 0.01 for comparison with CTR1 control group.

Oxaliplatin treatment did not significantly change the size profile of strongly ATP7A-expressing neurons, immunoreactivity pattern of ATP7A or CTR1, or the staining frequencies of ATP7A-or CTR1-positive cells (Figure 4A,B). In contrast, oxaliplatin treatment of animals caused atrophy of strongly CTR1-expressing DRG neurons, which showed a clear left-ward shift in their size distribution profile, reduction in their mean cell body areas from 1936 ± 278 μm^2 ^to 1461 ± 64 μm^2 ^(*P *< 0.01), a decrease in the percentage of large neurons measuring greater than 1750 μm^2 ^from 58.2 ± 16.1% to 28.5 ± 5.4% (*P *< 0.01), and an increase in the percentage of medium sized neurons measuring between 750 to 1750 μm^2 ^from 39.9 ± 15.9% to 66.4 ± 5.5% (*P *< 0.01) (Table 2).

## Discussion

This is the first description of the expression of copper-transporting P-type ATPases in DRG tissue from rats or any other animal species. Adult rat DRG tissue exhibited a specific pattern of expression of copper transporters with distinct subsets of sensory neurons intensely expressing either ATP7A or CTR1, but not both or ATP7B. Copper transporter mRNA levels in DRG were highest for CTR1, followed by ATP7A and barely detectable for ATP7B. ATP7A protein was detected in DRG tissue homogenates by Western blotting. ATP7A and CTR1 were detectable in DRG tissue by immunohistochemistry and were localised to the cell bodies of sensory neurons with little or no immunostaining of nerve fibres, satellite cells or other tissue elements, and without specific immunoreactivity for ATP7B. Neuronal immunoreactivity for ATP7A did not co-localise with CTR1, pNF-H or DAPI-stained satellite cells in double-label fluorescent immunohistochemistry studies, and their cell body size-profiles determined by morphometric analysis differed markedly from that of CTR1-immunoreactive neurons. Morphometric analyses of immunohistochemically defined subpopulations of cells in tissue sections is inherently subjective as qualitative interpretation of cell positivity and arbitrary definitions of positive and negative cells are required. However, strongly ATP7A-immunoreactive neurons accounted for about one third of the overall total population of DRG neurons, and were characterised by their small cell bodies and intense punctuate cytoplasmic immunostaining. In contrast, strongly CTR1-immunoreactive neurons accounted for about one tenth of the overall population of DRG neurons and were characterised by their large cell bodies, and intense plasma membrane and vesicular cytoplasmic immunostaining, as we recently described [35]. Together, these findings show that ATP7A and CTR1 have neuron subtype-specific and largely non-overlapping distribution in adult rat DRG tissue suggesting that these copper transporters have distinct roles in supporting the functions of primary sensory neurons.

The physiological significance of differential expression of copper transporters by DRG neurons is unclear and requires further study. However, ATP7A and CTR1 may be required by distinct sub-types of DRG neurons to deliver copper to specific cuproenzymes vital for the synthesis of neuropeptides and ATP. ATP7A, for example, delivers copper to peptidylglycine alpha-amidating monooxygenase in cell types other than DRG neurons [40-42], but this cuproenzyme activity is required by DRG neurons for the synthesis of substance P [10,43,44]. Like ATP7A, substance P is primarily expressed by small DRG neurons [26,45,46], and the size of substance P-expressing DRG neurons is not altered by oxaliplatin treatment [26]. This suggests the existence of a subset of sensory neurons that co-express ATP7A, peptidyl alpha-amidating monooxygenase and substance P to support neuronal functions requiring neuropeptide synthesis. In other cell types, the level of expression of CTR1 corresponds closely with the activity of cytochrome C oxidase [4,47,48], which is a cuproenzyme involved in oxidative phosphorylation ATP synthesis. Like CTR1, cytochrome C oxidase is expressed intensely by large-sized DRG neurons [8], which may have reduced capacity for glycolysis compared to small DRG neurons [49], consistent with their strong need for CTR1 to meet their high demands for copper delivery to cytochrome C oxidase and ATP synthesis via oxidative phosphorylation. In this way, the neuronal subtype-specific and largely non-overlapping distribution of ATP7A and CTR1 in DRG tissue may relate to specific cuproenzyme requirements by distinct subsets of primary sensory neurons.

Platinum antitumour drugs, such as oxaliplatin, are known for causing peripheral neuropathy by undefined mechanisms that might involve platinum accumulation within the DRG leading to atrophy or loss of peripheral sensory neurons [21-33,50-52]. Atrophy of DRG neurons would be expected to lead to altered sensory nerve conduction velocities that characterise oxaliplatin-induced peripheral neuropathy because DRG cell body size, axonal calibre and nerve conduction velocity are strongly correlated [53-55]. Furthermore, our previous work has suggested that oxaliplatin may induce atrophy of specific subpopulations of DRG neurons by causing the loss of phosphorylated neurofilament heavy subunit, which is a cyto-skeletal protein that determines the calibre of large myelinated DRG neurons and their axons [37]. In other cell types, the cellular accumulation and cytotoxicity of platinum drugs is controlled, at least in part, by copper transporters, with CTR1 transporting platinum drugs into cells [13-15], and ATP7A and ATP7B transporting platinum drugs out of cells or into specific subcellular compartments [16-20]. In the current study, we showed that oxaliplatin treatment of adult rats caused atrophy of the CTR1-immunoreactive subpopulation of DRG neurons without changing the size profile of the ATP7A-immunoreactive subpopulation of DRG neurons. It is possible that ATP7A expressing DRG neurons are less sensitive to oxaliplatin neurotoxicity because the high levels of ATP7A facilitate the cellular efflux of oxaliplatin reducing its availability for reactions with DNA or other key neurotoxicity targets. In contrast, DRG neurons expressing high levels of CTR1 would be expected to take up more oxaliplatin leading to toxic effects in this neuronal subtype. Thus we suggest the neuronal subtype-specific and largely non-overlapping distribution of ATP7A and CTR1 within DRG tissue influence the neurotoxicity of oxaliplatin by controlling its cellular accumulation and sub-cellular distribution within primary sensory neurons. If this is so, then oxaliplatin treatment could be expected to alter the expression, distribution and sub-cellular localisation of ATP7A and CTR1 as in other cell types [15,20,39,56,57] but no evidence for such a change was found in DRG tissue in the current study. The role of CTR1 and ATP7A in oxaliplatin neurotoxicity remains hypothetical but could be tested further in studies comparing the accumulation, sub-cellular distribution and neurotoxicity of oxaliplatin in CTR1-and ATP7A-expressing neuronal cells *in vitro *making use of a CTR1 inhibitor to block the neuronal uptake and neurotoxicity of oxaliplatin.

## Conclusions

In conclusion, adult rat DRG tissue exhibits a specific pattern of expression of copper transporters with distinct subsets of peripheral sensory neurons intensely expressing either ATP7A or CTR1, but not both or ATP7B. The neuron subtype-specific and largely non-overlapping distribution of ATP7A and CTR1 within rat DRG tissue may be required to support the differing cuproenzyme requirements of distinct subsets of sensory neurons, and could influence the transport and neurotoxicity of oxaliplatin.

## Methods

### Animals and drug treatment

Age-matched, 12-week-old female Wistar rats were housed in a self-contained unit maintained at 22 ± 2°C, and set to 12 h dark-light cycles with access to food and water *ad libitum*. Twelve healthy untreated animals were used for Cu transporter expression study by immunoblotting, immunohistochemistry and PCR respectively. In addition, for treatment study, two groups of animals received intraperitoneal injections of either oxaliplatin (Eloxatin; Sanofi-Aventis, Bridgewater, NJ, USA) at a dose of 1.85 mg/kg (n = 13) or dextrose (n = 12), as vehicle control, twice weekly for 8 weeks between 1300 and 1500 h. All animal procedures were approved by the institutional Animal Ethics Committee (AEC No. R591).

### Western blot analysis

Following euthanasia of animals with intraperitoneal injection of pentobarbitone (90 mg/kg body weight, Chemstock Animal Health, Christchurch, New Zealand), lumbar DRG tissues were dissected and homogenized using a Dounce homogenizer (Glas-Col, Terre Haute, IN, USA) for 3 min in a lysis buffer containing 250 mM sucrose, 1 mM EDTA, 1 mM EGTA, 0.5% NP-40, 0.1% SDS, and a protease inhibitor mixture (Complete Mini Protease Inhibitor Cocktail tablets; Roche Diagnostics, Indianapolis, IN, USA). The homogenate was centrifuged for 15 min at 500 ×*g *at 4°C to remove nuclei and large particulate matter, and the protein concentration of the resulting supernatant was determined by a bicinchoninic acid (BCA) assay as previously reported [58]. Protein samples (40 μg) were heated at 95°C for 30 min, resolved in 8% SDS-PAGE, and then transferred to a nitrocellulose membrane (Amersham Pharmacia, Tokyo, Japan) using a Transblot SD apparatus (Bio-Rad, Hercules, CA, USA). Following blocking with 5% milk/bovine serum albumin solution, ATP7A was detected by chemiluminescence using anti-ATP7A antibody (1:1000, no. ab13995: Abcam, Cambridge, UK), horseradish peroxidase (HRP)-conjugated anti-chicken antibody (Sigma-Aldrich, St. Louis, MO, USA), and the ECL Advance Detection reagent (Amersham Biosciences, Buckinghamshire, UK). Beta actin was probed to determine the equal loading using anti-beta actin antibody (Abcam) and a HRP-conjugated anti-rabbit IgG antibody (Amersham).

### Reverse transcriptase-PCR

Animals designated for *Atp7a*, *Atp7b *and *Ctr1 *RT-PCR analysis were euthanized with pentobarbitone as above described. The lumbar DRG, brain, spinal cord, liver, kidney and small intestine tissues were collected and homogenized in PureZol reagent for total RNA isolation using an Aurum Total RNA Fatty and Fibrous Tissue Kit (Bio-Rad). After digestion with DNase I (1 unit/μg, Bio-Rad), total RNA of each sample (0.25 μg) was reverse-transcribed into cDNA using a SuperScript first strand synthesis kit (Invitrogen, Carlsbad, CA, USA) according to instructions, followed by digestion with Ribonuclease H (Invitrogen) to remove the RNA templates. cDNA was amplified by PCR in a reaction mixture containing dNTP, MgCl_2_, Platinum *Taq *DNA polymerase (Invitrogen) and custom primers, using a GeneAmp 9700 PCR System (Applied Biosystems, Foster City, CA, USA) at 52°C for 40 cycles. Forward and reverse primers for rat *Atp7a *were: 5'-tag acg gca tgc att gta aat c-3' and 5'-tgg att tta cac ctg gct tct t-3'(amplicon of 375 bp); for rat *Atp7b *were 5'-att cca gga ctg tcc gtt cta a-3' and 5'-cac ttg ctc ctc tct gag gat t-3'(amplicon of 396 bp); for rat *Ctr1 *were: 5'-ttg gct tta aga atg tgg acc t-3' and 5'-cat aag gat ggt tcc att tgg t-3'(amplicon of 206 bp); and for rat glyceraldehyde-3-phosphate dehydrogenase (GAPDH): 5'-tgc tga gta tgt cgt gga gtc t-3' and 5'-aca gtc ttc tga gtg gca gta a-3' (amplicon of 291 bp), as a control. PCR products were electrophoresed in 2% agarose gel, stained with ethidium bromide and photographed using Gel Doc 2000 System (Bio-Rad).

### Real-time PCR

cDNA was synthesized from total RNA of lumbar DRG, brain, spinal cord, liver, kidney and small intestine tissues of healthy rats as above described, and used for multiplex real-time PCR using ABI PRISM 7900HT Sequence Detection Systems and SDS 2.3 software (Applied Biosystems). Primers and probe sets were obtained as TaqMan Gene Expression Assays containing forward and reverse unlabelled PCR primer pair and a fluorescent reporter dye-labelled TaqMan MGB probe (Invitrogen). Samples were analyzed in triplicate in a 10-μl total volume containing 25 ng of cDNA of each tissue, TaqMan universal PCR Master Mix, TaqMan FAM-labelled probes for rat *Atp7a *gene, *Atp7b *gene or *Ctr1 *gene, respectively, and VIC-labelled 18 S ribosomal RNA as endogenous control probe.

The abundance of mRNA of ATP7A, ATP7B, CTR1 or rRNA was measured as the threshold cycle values (Ct) after each reaction. Fluorescence values were plotted against cycle numbers in SigmaPlot 10.0 using sigmoidal 3 parameter fitting and 50% of the maximum fluorescence was taken as the Ct according to Liu at el's method [59]. The relative RNA expression level was calculated using the 2^-ρCt ^method [60], where gene of interest expression normalized to 18 S rRNA and ρCt = (Ct,_ATP7a or ATP7b or CTR1 _-Ct,_rRNA_).

### DAB and fluorescent immunohistochemistry of DRG

Animals were euthanized with pentobarbitone and perfused with phosphate buffered saline followed by 4% paraformaldehyde solution. Lumbar 5 DRG was dissected, post-fixated in the perfusion fixative for 2 h, cryoprotected in 30% sucrose overnight and embedded in Tissue-Tek (Sakura Finetechnical, Tokyo, Japan). Cryosections (12 μm) were thaw-mounted onto poly-L-lysine -coated Superfrost plus slides, rinsed, permeabilized in 0.2% Triton X-100, incubated with 1% hydrogen peroxide/methanol mixture (1:1), and blocked in 3% normal goat or donkey serum (Sigma-Aldrich) and 2% BSA (ICPbio Ltd, New Zealand). The slides were incubated with a chicken anti-ATP7A (1:1000; Abcam), a rabbit anti-ATP7B antibody (NB100-360, Novus Biologicals, Littleton, CO, USA) or a rabbit polyclonal anti-hCTR1 antibody (1:500, Novus Biologicals, Littleton, CO, USA), respectively, at room temperature overnight. Following rinses, the slides were incubated subsequently with a biotinylated secondary anti-chicken antibody (1:500, Jackson ImmunoResearch laboratories, PA, USA) or anti-rabbit antibody (1:500, Sigma-Aldrich) for 30 min, followed by an extravidin-peroxidase conjugate (1:500, Sigma-Aldrich) for 30 min. The peroxidase reaction was catalyzed using 3,3'-diaminobenzidine tetrahydrochloride (DAB) (AppliChem, Darmstadt, Germany) and hydrogen peroxide as substrates. The sections were dehydrated by gradient alcohols, cleared in xylene and coverslipped with DPX mounting medium. The negative control sections were processed by excluding the primary antibodies. Digital images were obtained using an Axiocam digital camera attached to an Axiostar light microscope and analyzed using Axiovision 3.0 software on a PC (Carl Zeiss, Hallbergmoos, Germany). For fluorescent double labelling, after blocking, incubation with 200 μl of Invitrogen Image-iT FX signal enhancer for 30 min and washes, DRG sections were incubated with the anti-ATP7A antibody (1:1000, Abcam), anti-hCTR1 antibody (1:1000, Novus) or anti-phosphorylated neurofilament heavy subunit (pNF-H) antibody (1:100, Swant, Bellinzona, Switzerland), respectively, at 4°C for 48 h, followed by subsequently Alexa Fluor 594-labeled anti-chicken or anti-mouse IgG, Alexa Fluro 488-labeled anti-rabbit IgG (1:500, Invitrogen), or DyLight 488-labeled anti-chicken IgG, at room temperature for 3 h. The sections were coverslipped with Vectorshield anti-fade mounting medium (Vector Laboratories, Burlingame, CA, USA). Reciprocal omission controls were included to ensure there was no cross-bleeding between the channels. Digital images were acquired using an Eclipse Ti fluorescence microscope with a cooled colour digital camera attached (Nikon, Japan), and analyzed using Nikon EclipseNet and ImageJ software (National Institutes of Health, USA).

### Morphometry

The size profiles of copper transporter-expressing DRG neurons were determined by measuring the staining frequency, mean cell body size and size distribution. Strongly ATP7A-expressing DRG neurons were defined as those having intense diffuse or punctuate cytoplasmic staining and/or plasma membrane immunoreactivity to ATP7A. Those negative for strong ATP7A expression had no or low-intensity diffuse or punctuate cytoplasmic staining without plasma membrane immunoreactivity. Strongly CTR1-expressing DRG neurons were defined as those having intense plasma membrane and/or punctuate cytoplasmic immunoreactivity to CTR1. Those negative for strong CTR1 expression had only diffuse cell body immunoreactivity without plasma membrane or punctuate cytoplasmic immunoreactivity. Between 1,041 and 1,586 neurons from every seventh tissue section were analyzed per DRG per animal for ATP7A and CTR1, respectively. The ATP7A-or CTR1-positive neurons were further arbitrarily categorized into three size-based groups: small ( < 750 μm^2^), medium (750-1750 μm^2^) and large ( > 1750 μm^2^), according to previous studies [26,61]. To determine the neurotoxicity of oxaliplatin, these morphometric parameters of ATP7A-positive and CTR1-positive subpopulations of DRG neurons were compared between the drug-treated and the control animal groups.

### Statistics

The differences in mean cell body size and staining frequency between different groups were assessed by one-way ANOVA with Bonferroni multiple comparison post test using Prism 5.01 software (GraphPad, San Diego, CA, USA), with a *P *value of < 0.05 indicating statistical significance.

## Competing interests

The authors declare that they have no competing interests.

## Authors' contributions

VI and JL carried out the experimental work. All authors contributed to the research plan, data interpretation and preparation of the manuscript.
